# Differentially private count queries over personalized-location trajectory databases

**DOI:** 10.1016/j.dib.2018.08.104

**Published:** 2018-09-03

**Authors:** Fatemeh Deldar, Mahdi Abadi

**Affiliations:** Faculty of Electrical and Computer Engineering, Tarbiat Modares University, P.O. Box 14115-194, Tehran, Iran

**Keywords:** Differential privacy, Count query, Personalized-location trajectory dataset, Benchmark dataset

## Abstract

Differential privacy is a technique for releasing statistical information about a database without revealing information about its individual data records. Also, a personalized-location trajectory database is a trajectory database where locations have different privacy protection requirements and, thus, are privacy conscious. This data article is related to the research article entitled “PLDP-TD: Personalized-location differentially private data analysis on trajectory databases” (Deldar and Abadi, 2018 [1]), in which we introduced a new differential privacy notion for personalized-location trajectory databases, and devised a novel differentially private algorithm, called PLDP-TD, to implement this new privacy notion. Here, we describe how the datasets in the research article were obtained and measure the relative error of PLDP-TD for different non-zero count query sets.

**Specifications table**

**Table:** 

Subject area	Computer science
More specific subject area	Information security; Data privacy; Differential privacy
Type of data	Table, figure, text file
How data was acquired	Processing of synthetic and real trajectory data
Data format	Raw, analyzed
Experimental factors	The continuous spatial domain was discretized by partitioning it into a uniform grid and then each grid cell was considered as a location. Then, the latitude-longitude coordinates in each input trajectory dataset were replaced by their corresponding locations. Afterwards, a privacy descriptor was assigned to each location, resulting in a personalized-location trajectory dataset.
Experimental features	A differentially private algorithm was applied to each personalized-location trajectory dataset and the average relative error of noisy answers to non-zero count queries was measured.
Data source location	N/A
Data accessibility	Data are with this article.

**Value of the data**

• The obtained personalized-location trajectory datasets can be used as benchmark datasets to examine the effectiveness of various differentially private algorithms developed for trajectory databases.
• The method used for obtaining personalized-location trajectory datasets can be used to obtain personalized datasets in other contexts.
• The personal privacy budget allocation strategy can be used as a reference strategy for effective non-uniform budget allocation in any tree-based structure.
• The results showed that by providing non-uniform privacy guarantees, the quality of noisy answers to count queries significantly improves.

## 1 Data

Our personalized-location trajectory datasets are obtained based on three synthetic and real trajectory datasets, namely, City80K [2], Geolife [3], and Taxi [4]. The City80K dataset simulates the routes of 80,000 citizens in a metropolitan area with 26 city blocks in 24 hours. The Geolife dataset collects the GPS trajectories of 182 users in Beijing, China, during a period of over five years (from April 2007 to August 2012), including not only common activities like go home and go to work, but also some entertainments and sports activities, such as shopping, hiking, and cycling. We choose the trajectories whose latitude-longitude coordinates (moving points) are between [39.4,40.8] latitude and [115.8,117.4] longitude, and break a trajectory if the interval time between its subsequent and current latitude-longitude coordinates is larger than one minute. Since each recorded trajectory consists of a sequence of latitude-longitude coordinates in a continuous spatial domain, we discretize the spatial domain by partitioning it into a 64×64 grid and then consider each grid cell as a location. We further replace the latitude-longitude coordinates by the obtained locations. Finally, the Taxi dataset contains approximately 5.2 million GPS trajectories of 8602 taxi cabs in Beijing, China, recorded during a 1-month period in May 2009. The trajectory data cover a region of Beijing restricted between [39.8,40.1] latitude and [116.1,116.6] longitude. Similar to the Geolife dataset, we discretize its spatial domain into a finite number of grid cells and then consider each grid cell as a location. However, due to smaller region size, we consider a 16×16 grid. Table 1 shows a summary of these trajectory datasets, providing information on the number of trajectories, the type of spatial domain, the number of locations, and the average and maximum trajectory lengths.

**Table 1:** Summary of trajectory datasets.

**Dataset**	**No. of trajectories**	**Type of spatial domain**	**No. of locations**	**Average trajectory length**	**Maximum trajectory length**
**City80K**	80,000	Discrete	26	4.52	24
**Geolife**	1,028,434	Continuous	4096	19.52	601
**Taxi**	5,251,604	Continuous	256	11.32	53,095

**Fig. 1:**
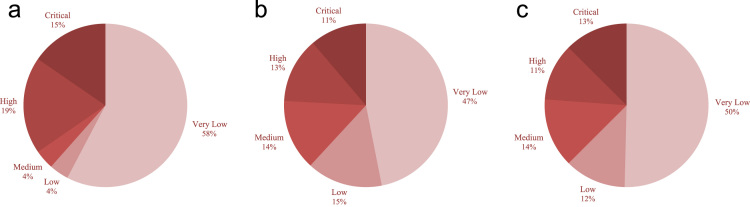
Distribution of privacy descriptors for different locations of the trajectory datasets: (a) City80K, (b) Geolife, (c) Taxi.

We randomly assign one of the five privacy descriptors, namely, Very Low, Low, Medium, High, and Critical, to each location of the trajectory datasets. We assume that the distribution of privacy descriptors of different locations in a trajectory dataset follows a Zipf distribution with shape parameter 0.50. We construct five non-zero count query sets on each trajectory dataset, each of which has different maximum query size (namely, 1, 2, 3, 4, and 5 for the City80K dataset; as well as, 2, 4, 8, 14, and 20 for the Geolife and Taxi datasets) and contains at most 10,000 randomly-generated count queries. It is important to note that by a non-zero count query set, we mean a set of count queries that inevitably exist in the original trajectory database. Figs. 1a to c show the distribution of privacy descriptors for different locations of the trajectory datasets.

## 2 Experimental design, materials and methods

Personalized-location differential privacy (PLDP), proposed in our research paper [1], is a new differential privacy notion for trajectory databases that relaxes privacy guarantee for trajectory data records with low privacy levels in order to improve the utility of data analysis. In fact, by introducing this concept, data owners are allowed to take the privacy protection requirements of different locations into consideration. Based on this new privacy notion, we have devised a differentially private algorithm, called PLDP-TD [1], which allocates a personal privacy budget to each trajectory data record based on the privacy protection requirements of locations it contains. PLDP-TD employs two different strategies for personal privacy budget allocation: non-adaptive and adaptive. In this data article, we first compare the impact of these strategies on the average relative error of noisy answers to count queries.

Table 2 reports the average relative errors (in percent) of PLDP-TD with non-adaptive and adaptive strategies for different non-zero count query sets on City80K, Geolife, and Taxi under different total privacy budgets. From the table, we can observe that the average relative error of the adaptive strategy is lower than that of the non-adaptive one. This is due to the fact that, the adaptive strategy tries to make effective use of privacy budgets. Therefore, we continue to experiment with this strategy. From now on, for convenience, we refer to PLDP-TD with adaptive strategy simply as PLDP-TD.

**Table 2:** The average relative errors (in percent) of PLDP-TD with non-adaptive and adaptive strategies for different non-zero count query sets on City80K, Geolife, and Taxi under different total privacy budgets.

**Algorithm**	ε	**City80K**					**Geolife**					**Taxi**				
		**1**	**2**	**3**	**4**	**5**	**2**	**4**	**8**	**14**	**20**	**2**	**4**	**8**	**14**	**20**
	**0.05**	123.60	63.42	62.89	64.92	63.09	48.90	52.70	59.67	54.32	54.56	21.19	20.38	21.46	22.16	20.56
**Non-adaptive**	**0.10**	82.70	44.25	42.89	44.31	43.26	31.62	34.47	37.66	33.83	34.35	19.65	18.61	19.53	20.04	19.04
	**0.50**	59.03	32.73	31.20	31.30	30.78	19.10	20.65	20.47	18.29	19.07	18.66	18.11	18.69	18.97	18.32

	**0.05**	109.71	57.78	56.58	58.61	56.31	48.74	52.58	59.47	54.17	54.45	20.10	19.35	20.37	20.84	19.61
**Adaptive**	**0.10**	75.64	40.99	39.61	40.67	39.61	31.42	34.36	37.49	33.67	34.20	18.58	17.63	18.47	18.86	18.01
	**0.50**	57.58	31.85	30.39	30.44	29.93	18.59	20.04	20.02	17.94	18.55	18.29	17.71	18.30	18.47	17.96

**Fig. 2:**
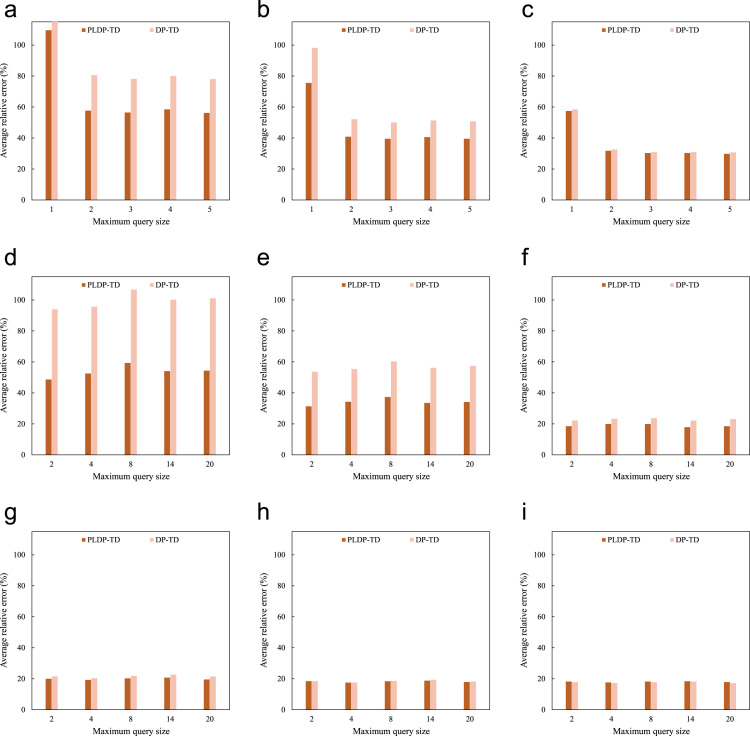
The average relative errors (in percent) of PLDP-TD and DP-TD for different non-zero count query sets on City80K, Geolife, and Taxi under different total privacy budgets: (a) City80K, ε=0.05, (b) City80K, ε=0.10, (c) City80K, ε=0.50, (d) Geolife, ε=0.05 (e) Geolife, ε=0.10, (f) Geolife, ε=0.50, (g) Taxi, ε=0.05, (h) Taxi, ε=0.10, (i) Taxi, ε=0.50.

In continue, we compare the average relative error of PLDP-TD in the normal case with the case when all locations of the underlying geographical map have the same privacy descriptor “Critical”. When applying PLDP-TD to the second case, we refer to it simply as DP-TD. Figs. 2a to i compare the average relative error (in percent) of PLDP-TD with that of DP-TD for different non-zero count query sets on City80K, Geolife, and Taxi under different total privacy budgets. Obviously, we can observe that the average relative error of PLDP-TD is significantly smaller than that of DP-TD, which is the result of relaxing privacy guarantee for trajectory data records that have low privacy levels.
